# Impact of pornography consumption on children and adolescents: a trauma-informed approach

**DOI:** 10.3389/frcha.2025.1567649

**Published:** 2025-09-29

**Authors:** Mar Alvarez-Segura, Ines Fernández, Yousra El Kasmy, Esther Francisco, Sonsoles Gallo Martínez, Eva Maria Ortiz Jiménez, Anna Butjosa

**Affiliations:** ^1^ Department of Child and Adolescent Mental Health, Hospital Sant Joan de Déu, Esplugues de Llobregat, Spain; ^2^ Department of Psychology, CEU Abat Oliba University, Barcelona, Spain; ^3^ Child and Adolescent Mental Health Research Group, Institut de Recerca Sant Joan de Déu (IRSJD), Esplugues de Llobregat, Spain; ^4^ Department of Child and Adolescent Mental Health, Bellvitge University Hospital, Barcelona, Spain; ^5^ CEU Schools Area, Fundación Universitaria San Pablo CEU, Madrid, Spain; ^6^ Center for Biomedical Research in Mental Health Network (CIBERSAM), Madrid, Spain

**Keywords:** children, adolescents, pornography, sexual violence, trauma

## Abstract

Parallels may exist between consequences of underage pornography use and the post-traumatic symptoms of child sexual abuse. Could pornography alter child and adolescent development and become a trauma in itself? Child victims of these images could face a conflict similar to witnesses of domestic violence, but instead of impacting mainly on the bonding system, it would affect the sexual system. Victims faced with the erotisation of violence are subjected to contradictory, incomprehensible, and sometimes inexplicable forces, which can lead to a traumatised sexuality with negative consequences in interpersonal relationships. The inability to explain something, or to make sense of it, activates the three classic pathways of trauma. One response to trauma is flight, which can lead to distancing from oneself and from others. This isolation, moreover, reinforces the consumption of pornography. Another response is the struggle to overcome the impact through self-control and aggression. Sexual coercion may appear as an attempt to modulate one's own contradictory emotions, as a form of self-protection and avoidance of the dreaded humiliation. Finally, there may be a dissociation response in the re-victimisation that appears in affected children. Unable to find a way to integrate the scenes, these minors may end up learning to adopt a posture of absolute surrender. The reconceptualisation of pornography in underage consumers as something potentially traumatic would help to better our understanding of its effects and the differing susceptibility of the victims, so that we may develop real and effective legislation and more appropriate therapeutic interventions.

## 1 Introduction

Access to internet pornography by children and adolescents has increased so significantly in the last decade (1) that it can be considered a public health problem (2). This circumstance is further exacerbated by ease of access: 90% of pornography consumption occurs via mobile devices (3). Although the Diagnostic and Statistical Manual of Mental Disorders (DSM 5-TR) does not formally recognise internet use disorders, the American Psychiatric Association acknowledges these disorders as a growing concern that includes problematic internet pornography use (4).

In response to this reality, recent years have seen an increase in studies addressing this problem (5–10) with a focus on the association between pornography and sexual aggression in children and adolescents (11). However, reviews have not reached consistent conclusions regarding the relationship between pornography and the sexual attitudes and behaviours of minors. This inconsistency is largely due to methodological challenges, such as that of obtaining sensitive information from underage participants and ethical constraints that render experimental studies impossible. What is consistently confirmed, however, is the link between sexual violence and the consumption of violent pornography (12, 13), as well as its impact on aggressive behaviour in individuals with low scores in agreeableness—colder, more hostile, and distrustful personalities (14). This article does not aim to confirm the negative effects of pornography on minors, which would require a systematic review. Instead, it proposes to point out that the negative effects of pornography consumption in minors described in the literature have a symptomatic parallel with the impact of trauma. For this reason, articles published in the last decade that present negative effects of pornography consumption in the child and adolescent population are selected.

In the field of traumatology, a traumatic event is defined as one that exceeds an individual's usual coping capacity. Such events are perceived as meaningless and threatening because the usual resources for managing new situations prove insufficient. When individuals lack a framework to contextualise the event they are experiencing, it disrupts the meaning of their own experience. Peter and Valkenburg (9) define internet pornography as professionally produced or user-generated images or videos intended to sexually arouse the viewer. The nature of pornography, which often lacks exploration, rituals, restrictions, or even consent, makes its content potentially violent. Pornographic platforms are optimally designed to capture children and adolescents with awakening healthy sexual curiosity. For those whose sexual selves are still developing, these images are difficult to interpret accurately and may trigger survival responses.

Many of the symptoms observed in minors exposed to pornography parallel post-traumatic symptoms. This raises the following question: can pornography disrupt child and adolescent development and become a trauma in itself? The impact of pornographic content often exceeds minors' processing capacity, resulting in a traumatic effect evidenced by the psychological consequences of consumption. Recent studies of minors who are victims of technology-facilitated sexual abuse show negative consequences strikingly similar to those experienced by sexually abused minors (15). This prompts further inquiry: could minors consuming pornography also behave like witnesses of violence? This article aims to highlight the parallels between the consequences of pornography consumption in minors and post-traumatic symptoms. Due to lack of empirical evidence, it would be premature to affirm this relationship, however, the possible association is described. Reconceptualising pornography as potentially traumatic could provide a deeper understanding of its effects and the differing susceptibility of minors according to their age, paving the way for more effective and realistic legislation and more appropriate therapeutic interventions.

## 2 Impact of pornography consumption on minors' mental health

A study conducted across six European countries found that 54% of adolescents are exposed to online pornography, with 24% consuming it weekly (5). Nationally representative surveys of adolescents in the USA have found that 68.4% reported exposure to online pornography (16). A 2016 Spanish study involving a population ranging from 13 to 17 years old found that 60% of boys and 11% of girls used the internet for sexual activities (17). At the same time, 75% of parents believe their children have never been exposed to pornography (18).

The explicit images, underlying messages, normative symbolic nature, and sequence of sexual behaviours depicted in pornography can influence the emotional, cognitive, and behavioural aspects of sexuality, particularly when these aspects are not yet well established (19). Similar to the impact of excessive media use, pornography consumption can lead to a time displacement effect. Instead of spending time in cognitively stimulating activities such as completing homework or studying, adolescents allocate more time to viewing internet pornography. As a result, students' academic performance tends to deteriorate (20).

Prolonged exposure to pornography is known to lead to habituation, resulting in blunted processing of pleasurable stimuli and greater sensitivity to negative stimuli (21). Continuous use of pornography impairs emotional processing capacity and flattens affect, reducing emotional connection to real-life sexual experiences. The reward and gratification system adapts by releasing large amounts of dopamine, but tolerance develops, requiring increasingly higher doses, quantities, and intensity to achieve arousal. This raises the dopamine—or pleasure—threshold so high that real-life experiences fail to provide the necessary pleasure. A study of university students found that low levels of cognitive and affective empathy were associated with heavy consumption of pornography and higher prevalence of risk behaviours (22). Other studies suggest that pornography consumption can influence the development of sexual attitudes and behaviours in adult and young people (23, 24).

According to Simon and Gagnon's sexual script theory, frequent exposure to sexually explicit scenes in media can affect young people's understanding of sexuality and the development of sexual scripts (14). Pornography depicts sexual relations in a stereotypical manner, from beginning to end, prominently featuring aspects of sexuality grounded in male dominance while perpetuating sexism (25) and explicitly depicting coercion, exploitation, and violence (26). In the pornographic depiction of sex, there is no elaboration of emotions, and the viewer is unable to perceive the connection between sex and intimacy (27).

All this may have a greater impact on children and adolescents. The prefrontal cortex is responsible for impulse control, and during adolescence it is still under maturation, which makes youngsters more inclined toward sensation-seeking and risk-taking behavior. In addition, children's naivety and less developed self-reflection ability complicate their capacities to set boundaries (15). In a U.S. survey of college students, 12% of boys and 18.7% of girls indicated that viewing pornography before the age of 18 had a strong emotional impact, with over two-thirds reporting feelings of surprise or shock, and around half of the boys and one-third of the girls experienced guilt or shame (28). Egodystonia is observed in adolescents who perceive a contrast between their idealised notions of love and the sexual humiliation and explicitness portrayed in pornography.

It is worth mentioning that despite this, concerning adolescents (years 11–19) longitudinal findings (29, 30) often fail to confirm broad harms on permissiveness, sexual satisfaction, or general mental health. Adolescents' reactions to pornography consumption largely depend on the context, age, type of content, mode of exposure, and the individual characteristics of the child or adolescent. This points to a clear need for additional empirical investigation within this demographic group.

## 3 Pornography and sex differences

Accessing pornography appears to be predominantly a male activity (31–33). Men tend to report more problematic pornography use (PPU) than women, and sexual minority men and women tend to report more PPU than heterosexual men and women (34).

These sex differences were also evident in a report by Villena-Moya et al. (35) which showed that adolescent males demonstrated higher levels of intentional pornography use and PPU, with sexual pleasure as a central variable. For females, however, online victimization and loneliness emerged as key factors, highlighting their vulnerability to digital harm. A study by Ballester et al. (36) indicates that 90.5% of adolescents aged 13–18 reported watching pornography in recent years (91.7% of male adolescents and 89.3% of female adolescents). Notably, the study points up similar ages of initial exposure, indicating a trend toward convergence in the age of initiation between sexes, although with differing motivation.

Regarding interactional style, a pattern emerges in which men are typically portrayed in dominant roles, while women are often depicted as passive objects within the relationship (31, 37). PPU in males is associated with more sexist models of sexuality in adolescents (35). Male dominance in sexual relationships is stereotypically reinforced, accompanied by the perpetuation of rape myths and sexual beliefs, driven by relationships (38, 39). Among men, a positive correlation exists between a craving for pornography and sexist beliefs, driven by the violent and objectified portrayal of women in pornography (25). Additionally, men who consume pornography are more prone to engaging in sexual coercion (40, 41).Women, usually depicted in pornography as acting to satisfy their male partners' desires, are often portrayed as sexual objects subjected to humiliation and domination (42). Individuals consuming pornography internalise not only the sexual behaviours displayed but also the patterns and stereotypes embedded in their intrapsychic scripts (43), which become further reinforced through continued consumption. The social learning theory of Bandura provides a framework for understanding this phenomenon, as reinforcement occurs due to the visualization of representations of rewards when participating in sexual behaviours.

Escalations in violent behaviours within interpersonal relationships have been identified. This escalation of behavior, including sexual assault and subsequent victimisation through relationship violence, is linked to early exposure to pornography, particularly with violent content (8, 13).

## 4 Pornography consumers as witnesses of sexual violence

This final section explores the intersections between trauma and pornography, as well as the similarities between the effects of childhood sexual violence and the disorders linked to pornography consumption in children and adolescents.

The first comparison concerns viewing pornography and traumatic events. According to the DSM-5R, sexual trauma includes witnessing sexual violence. For children, witnessing sexually violent events constitutes an experience inappropriate for their development stage, even when these events do not involve physical violence or injuries (44). This perspective positions pornography consumption as an experience that is potentially inappropriate and traumatic for development. At early ages, children lack the capacity for critical thinking and the ability to discern what is good or harmful, further underscoring the developmental risks associated with exposure.

A common element in trauma definitions is the focus on an individual factor, succinctly captured in van der Kolk's definition (45): trauma occurs when internal and external resources are insufficient to cope with an external threat. Viewing of pornography by minors aligns with this understanding of trauma, as it overwhelms their capacity to process what they see, disrupts their frames of reference (46), and distorts the basic schemes that help them understand and adapt to the world (47).

A second shared element of pornography and traumatic events is the disturbing nature of the content. A defining characteristic of a traumatic event is its adverse, threatening, and unexpected nature. What should unfold as a gradual, comprehensible process of sexual development does not occur. Painful or frightening emotions become traumatic when they lack a context of emotional understanding in which they can be integrated—a relational space for containment (48). In such cases, feelings of alienation and loneliness take shape. Finally, the impact of pornography consumption can be reinterpreted though a trauma model, highlighting symptomatic coincidences. Van der Kolk (45) identifies three types of maladaptive behaviour: actions that harm others, self-destructive behaviours, and revictimization. Promiscuous, dominant, violent, or submissive sexual behavior may correspond to these trauma responses.

**Figure 1:**
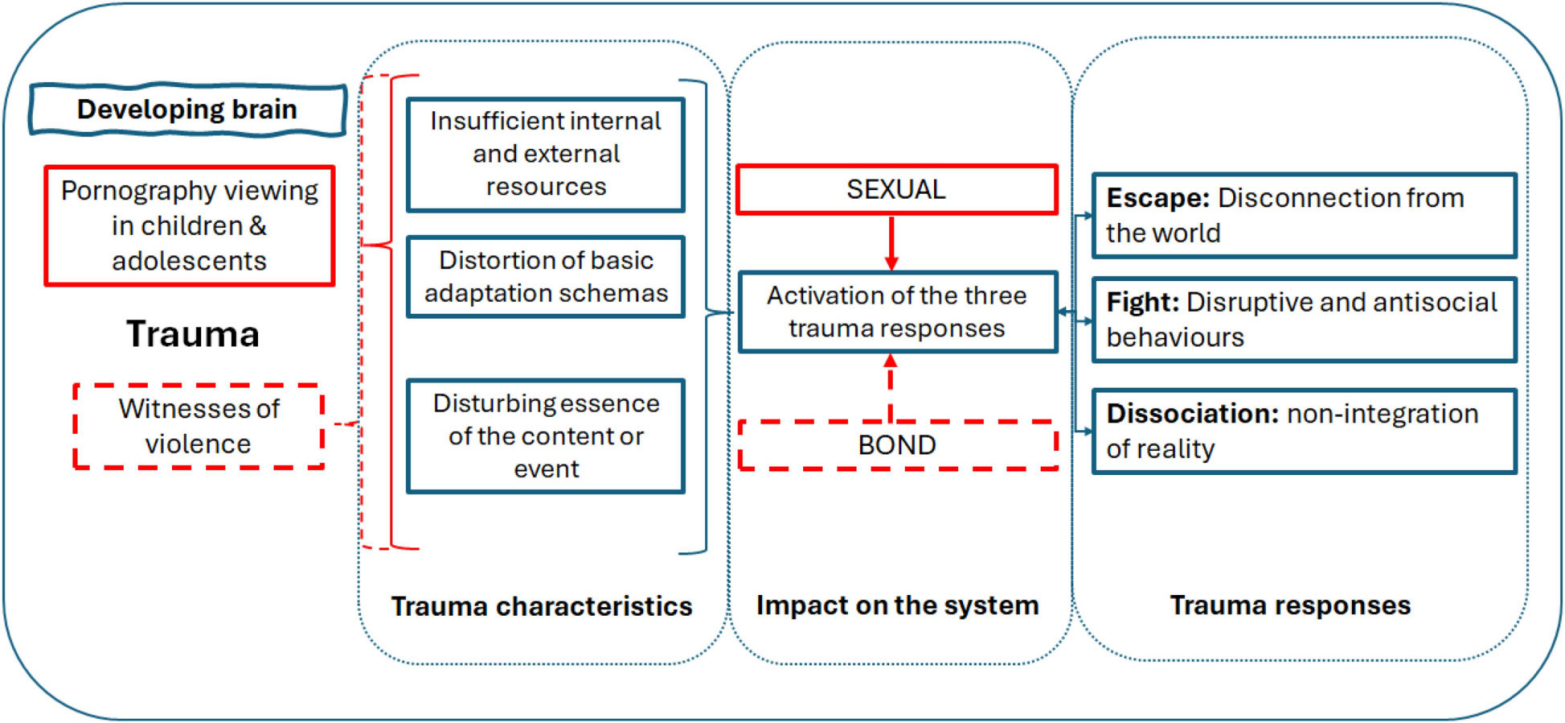
Diagram of the characteristics of trauma in the sexual and bonding system.

Minors exposed to these images face a conflict like that experienced by witnesses of domestic violence (see Figure 1). However, instead of impacting the bonding system, it affects the sexual system. These minors find themselves caught between sexual attraction and curiosity on the one hand, and the shock of explicit sexual images on the other. The excitement caused by these images may spark genuine interest in sexual discovery but is often accompanied by fear, anger, or even disgust. The victim, confronted with the eroticisation of violence, is subjected to contradictory, incomprehensible, and sometimes inexplicable forces, which hinders sexual integration. This difficulty is heightened during adolescence, as the activation of sexual steroids plays a critical role in the biological mechanisms of both sexual and aggressive behaviours. In males, in particular, learning to inhibit aggressive behaviours in sexual contexts is crucial (49). When a minor is unable to make sense of or integrate the perceived images, it can activate the three trauma pathways. An imbalance between the impulsive and reflective system has been detected in males with a tendency toward internet-pornography use disorder (50). Just as the safety of bonded relationships allows for emotional regulation and confident exploration—essential to personal development—the safety of sexual experiences similarly fosters gradual and confident exploration that helps regulate sexual feelings. However, sexuality traumatised by pornography is unlikely to find the appropriate channels for integration, potentially triggering the three classic trauma responses, which will impact both the sexual system and its meaning.

One response to trauma is flight, which can lead to protecting oneself, disconnecting from the world, and distancing from oneself and others (46). Similar to the effects of child sexual abuse, minors who consume pornography may experience difficulties in trusting others, alongside a tendency to feel emotionally isolated and in addictive stages away from others. This isolation, in turn, reinforces pornography consumption, creating a vicious circle (51).

Another response is fight, which may manifest as an attempt to overcome the impact of trauma through self-control and aggression. A population-based cohort study spanning 50 years showed that child maltreatment is associated with an increased risk of antisocial behaviour (52). Developing antisocial behaviour could occur as a form of self-protection, aimed at avoiding the deeply feared experience of humiliation. Sexual coercion can appear as an attempt to modulate one's own contradictory emotions (53). This response is often accompanied by negative distortions of omnipotence. The escalation of the feeling of omnipotence allows a shift from perceiving desires as personal wishes to framing them as entitlements. This distorted perception of reality plays a significant role in the escalation of problematic sexual behaviour and is reinforced by emotional blunting. In this sense, this distorted perception is compounded by the current use of artificial intelligence in the generation of pornographic content. While individuals may rationalise their actions by thinking “*it's digital, not real*”, the critical reflection is that although the image may be digital, the harm is real because the person behind it is real.

The existing evidence points toward greater consumption of sexualized media is associated with a greater likelihood of sexual coercion perpetration and victimization in sexually active adolescents. More specifically, when adolescents viewed online pornography and sexually oriented reality television more frequently, they had an increased likelihood of displaying sexual coercion perpetration (54, 55).

Depending on the characteristics of the minor and the content of the pornography, viewing it may lead to a final response of dissociation. Dissociation involves an attempt to deny that an intolerable situation is occurring or that the individual is present in that situation (56). Of particular concern in this context is the revictimisation inherent in child sexual abuse. Greater severity of dissociation during sex has been linked with greater sexual dysfunction and higher compulsive sexual behavior disorder in childhood sexual abuse survivors in adulthood (57). Female minors who consume pornography may also resort to dissociative mechanisms as a means of escaping the violation of their gaze. The dominant feeling of helplessness and sexual humiliation witnessed in such content could trigger this response in females. Unable to find a way to process and integrate the scenes, they may ultimately learn to adopt a stance of absolute surrender. Family and social environments where relationships of care and respect have been deeply violated can further intensify this response.

Childhood sexual abuse, like pornography consumption, is associated with negative self-perceptions, including low self-esteem, detrimental and pervasive shame (58) and guilt, as well as cognitive distortions and a tendency toward self-mutilation (46). The cognitive component of guilt reflects the individual's perceived role in the event, encompassing feelings of having acted wrongly, responsibility for causing the event, lack of justification, and erroneous beliefs about prior knowledge of the outcome (59). These perceptions contribute to revictimisation in various forms.

Finally, it is worth redefining the reasons for the impairment of attention in minors who consume pornography. Undoubtedly, their attention is disrupted by the time spent in online consumption itself, as well as the cognitive absorption it induces (60). To these factors, we should add the excitation transfer model (61), which posits that physiological activation does not dissipate immediately after the conditions that caused it have ended but rather takes time to disappear. Thus, the excitement caused by the pornography consumption in minors can carry over into the academic context and hinder emotional regulation and attention. In addition to these models, attention and concentration could also be affected by trauma. In such cases, the disruption stems from a dissociative process that leads to a withdrawal into oneself, as if an internal concern or emotional state were interfering with the ability to remain connected with tasks and connected to others.

## 5 Conclusions

Many of the psychological consequences observed in young pornography consumers could align with post-traumatic symptoms experienced following childhood sexual abuse. The culture of pornography and its normalisation can lead to our overlooking the significant impact it has on minors, especially at an age when their sexual self is still undeveloped, leaving them without the tools needed to decode the eroticisation of violence.

Social learning is crucial to understanding the behaviours and symptoms that may arise in minors. However, what is even more concerning is that these symptoms may reflect a traumatized sexuality that, in desperate attempts at integration, defaults to post-traumatic survival responses. This reality would complicate the treatment of affected minors, as their symptoms could not be not merely the result of learned behaviours or conditioning but rather stem from a traumatic disintegration of their sexual system.

Future empirical research should be done in order to analyze the possible effects of pornography consumption on childreńs symptoms. As clinical trials have ethical limitations, path analysis could be carried out for this purpose. The combined role of different potential mediators as negative life events, lack of social support or abuse could provide a better understanding of the impact of pornography consumption on post-traumatic symptoms in children and adolescents. In this context, protective factors as the role of caregivers and education become essential.

Active involvement in the prevention of and education about pornography use can mitigate these effects. There are approaches that combine comprehensive sexual education with porn-literacy skills [e.g., (62)]. However, the purported benefits of integrating comprehensive sexual education with porn-literacy skills in adolescents remain subject to debate. There is currently insufficient consensus regarding its effectiveness, and further empirical research is required to evaluate its impact across diverse developmental and sociocultural contexts (63).

The beneficial effects of pornography consumption in children and adolescents have not been empirically demonstrated. Nonetheless, unrestricted access to such content on the internet was not initially questioned and remains largely unregulated in most countries. Implementing protective measures for children and adolescents in relation to pornography consumption requires rigorous research—research that, notably, has not been demanded to justify the widespread and indiscriminate access currently available to this population. While ongoing studies aim to produce more conclusive findings, UNICEF has issued clear recommendations on the matter: “Pornographic content can harm children. Exposure to pornography at a young age may lead to poor mental health, sexism and objectification, sexual violence, and other negative outcomes. Among other risks, when children view pornography that portrays abusive and misogynistic acts, they may come to view such behaviour as normal and acceptable” (64).

Studies emphasize that open and direct communication between parents and children reduces positive attitudes toward pornography (65). According to Rasmussen et al. (66), this reduction is associated with lower consumption during adolescence and adulthood, thereby contributing to the healthier development of the sexual system.

It is crucial to underscore that traumatic reactions resulting from pornography consumption in minors may vary depending on the context, age, type of content, mode of exposure, and the individual characteristics of the child or adolescent. These reactions are even more likely when the material involves violent, degrading, or coercively eroticized content. Therefore, it is necessary to define, as with all types of childhood trauma, the characteristics of the risk factors that make a child more vulnerable to suffering post-traumatic stress from pornography consumption, and the varying degrees of minor susceptibility, as well as protective factors.

But most importantly, there is a need to establish and implement effective legislation to regulate it. While many jurisdictions have successfully restricted childreńs access to pornography contents in non-digital media, efforts to achieve similar restrictions in the digital environment have proven ineffective (64).
